# N‐α‐Acetyltransferase 10 inhibits invasion and metastasis of oral squamous cell carcinoma via regulating Pirh2‐p53 signalling pathway

**DOI:** 10.1111/jcmm.17306

**Published:** 2022-04-02

**Authors:** Fazhan Wang, Jun Zheng, Jie Yang, Ting Luo, Jiang Xu, Yongyong Yang, Yongqing Gu, Yan Zeng

**Affiliations:** ^1^ Precision Clinical Laboratory Central People's Hospital of Zhanjiang Guangdong Medical University Zhanjiang Central Hospital Zhanjiang China; ^2^ Key Laboratory of Xinjiang Endemic and Ethnic Disease School of Medicine Shihezi University Shihezi China; ^3^ 12244 Department of Urology Northwestern University Feinberg School of Medicine Chicago Illinois USA; ^4^ 96702 Beijing Key Laboratory for Radiobiology Beijing Institute of Radiation Medicine Beijing China

**Keywords:** invasion, metastasis, NAA10, OSCC, Pirh2‐p53

## Abstract

N‐α‐Acetyltransferase 10 (NAA10) was reported to be involved in tumour invasion and metastasis in several of tumours. However, the role and mechanism of NAA10‐mediated invasion and metastasis in oral squamous cell carcinoma (OSCC) remains undetermined. Herein, our study showed that NAA10 inhibits cell migration and invasion in vitro and attenuates the xenograft tumorigenesis in nude mice. Mechanistically, we demonstrated that there is a physical interaction between NAA10 and RelA/p65 in OSCC cells, thereby preventing RelA/p65‐mediated transcriptional activation of Pirh2. Consequently, inhibition of Pirh2 increased p53 level and suppressed the expression of p53 downstream targets, matrix metalloprotein‐2 (MMP‐2) and MMP‐9. Therefore, NAA10 may function as a tumour metastasis suppressor in the progression of OSCC by targeting Pirh2‐p53 axis and might be a prognostic marker as well as a therapeutic target for OSCC.

## 1 INTRODUCTION

Oral squamous cell carcinoma (OSCC), a subset of head and neck squamous cell carcinoma (HNSCC), is the most prevalent malignant neoplasm of the oral cavity,^1^ with approximately 300,000 newly diagnosed cases and 170,000 cancer deaths worldwide each year.^2^ Although substantial development in both the diagnosis and sequential treatment over recent decades, the long‐term survival of OSCC patients has not been significantly improved. One of the most common causes that lead to death in OSCC patients is invasion and metastasis. The 5‐year survival rate with vascular and perineural metastasis is 40% and 47%, respectively, while the 5‐year survival rate with distant metastasis is only 29%.^3^ However, the molecular mechanism underlying the metastasis of OSCC remains largely unknown.

N‐α‐Acetyltransferase 10 (NAA10/ARD1), the catalytic subunit of N‐terminal acetyltransferase complex (NatA), has both N‐α and N‐ε acetylation activities.^4^, ^5^, ^6^ NAA10 is involved in regulating cell proliferation,^6^, ^7^, ^8^ apoptosis,^9^, ^10^ autophagy,^11^, ^12^ tumour metastasis^13^, ^14^ and cell cycle arrest.^15^ NAA10 overexpression has been documented in breast cancer,^16^ colorectal cancer,^17^ hepatocellular cancer^18^ and lung cancer.^19^ While downregulation of NAA10 in thyroid neoplasm,^20^ non‐small cell lung cancer (NSCLC)^11^ was also reported. Although the efforts to elucidate the biological function of NAA10, it remains disputed regarding its roles in cancer. NAA10 was shown to physically interact with and acetylated the androgen receptor (AR) and form a positive feedback loop for AR‐dependent prostate tumorigenesis.^8^ Moreover, in lung cancer cells, NAA10 potentiates DNMT1’s affinities with promoter regions of tumour suppressor gene E‐cadherin and LATS, thereby suppressing their transcription and facilitating tumorigenesis.^21^ NAA10 was also reported to interact with β‐catenin and promote transcription of cyclin D1 in lung cancer cells.^6^ By binding to p65 subunit of nuclear factor‐κB (NF‐κB), NAA10 may increase MCL1 transcription and resistance to stimuli‐induced apoptosis.^10^ In osteosarcoma, NAA10 was directly associated with MMP‐2 protein through its acetyltransferase domain and maintained MMP‐2 protein stability via NatA complex activity.^14^ However, other studies found that NAA10 may serve as a tumour suppressor. By binding to PIX proteins, NAA10 inhibited Cdc42/Rac1 activity and, therefore, suppressed tumour metastasis.^19^ Besides, NAA10 inhibits the migration and invasion of breast cancer cells by binding to STAT5a and decreases STAT5a‐stimulated ID1 expression.^13^ TSC2 was found to be acetylated and stabilized by NAA10, through which NAA10 inhibits mammalian target of rapamycin signalling pathway and suppressed tumorigenesis.^11^ NAA10 may play diverse roles in different types of cancer cells or different stages during cancer tumorigenesis, and thus, identifying cancer‐type‐specific targets will help to understand the role of NAA10 in a certain cancer type.^14^

p53 has been shown to involved in the regulation of cell cycle arrest, DNA damage repair and apoptosis.^22^, ^23^ Of particular interest, the emerging evidence demonstrated that p53 plays a critical role in inhibiting cancer invasion and metastasis.^24^, ^25^ Matrix metalloproteinases (MMPs) play an important role in tumour invasion, metastasis and tumour‐induced angiogenesis by degrading basement membrane and extracellular matrix (ECM).^26^, ^27^ It has been shown that p53 can potently attenuate the expression of MMP‐1,^28^ MMP‐2,^29^ MMP‐9^30^ and MMP‐13.^31^ Pirh2 is a p53‐induced protein, and has been shown to ubiquitylate p53 in vivo and in vitro.^32^ Indeed, Pirh2, as an oncoprotein, was found to be stabilized and upregulated in several of tumour tissues, including head and neck cancer.^33^ The overexpression of Pirh2 was concomitant with decreased p53 levels in malignant tissues, suggesting a role for Pirh2 in tumorigenesis through regulation of p53 stability and expression.^34^

In previous study, we revealed that the expression of NAA10 was negatively correlated with that of Pirh2 in OSCC tissues. Besides, positive NAA10 and negative Pirh2 might be independent biomarkers for better prognosis in OSCC patients.^35^ However, the precise mechanism that NAA10 downregulated Pirh2 remains unknown. Here, we demonstrated that NAA10 plays a role in the invasion and metastasis of OSCC. Mechanistically, we elucidated that NAA10 interacts with RelA/p65 in the cytoplasm of OSCC cells, and subsequently inhibits RelA/p65‐mediated transcriptional activation of Pirh2 by preventing the nuclear translocation of RelA/p65. Consequently, inhibition of Pirh2 increased p53 level and suppressed the expression of p53 downstream targets, MMP‐2 and MMP‐9. These data revealed that NAA10 may function as a tumour metastasis suppressor in the progression of OSCC by targeting Pirh2‐p53 axis and might be a prognostic marker as well as a therapeutic target for OSCC.

## 2 MATERIALS AND METHODS

### 2.1 Cell lines, animals and reagents

Human oral squamous cell carcinoma cell lines CAL 27 and SCC‐15 were obtained from ATCC and cultured with standard culture conditions. Cell line authentication was performed according to United Kingdom Coordinating Committee on Cancer Research Guidelines every 2–3 months, including mycoplasma test by PCR and measurement of cell proliferation by counting. Female BALB/c nude mice, 6 weeks old, were purchased from the Beijing Laboratory Animal Center (Beijing, China). Primary antibodies used in this study were listed in Table S1.

### 2.2 Plasmids, small interfering RNAs, transfection and stable cell line generation

Human full‐length pCMV‐RelA/p65 plasmid and the plasmids with silencing and overexpressing NAA10, shCon/shNAA10 and pcDNA3.1/ pcDNA3.1‐NAA10 were kindly donated by professor Chengchao Shou (Peking University Cancer Hospital & Institute). The luciferase plasmid pGL3‐Pirh2 was constructed from upstream 1763 bp of the human *Pirh2* gene transcription start site to downstream 144 bp of the gene transcription start site (1907 bp). At the same time, pRL‐TK Renilla luciferase plasmid was purchased from GenePharma. Small interfering RNAs (siRNAs; listed in Table S2) were synthesized by GenePharma. RNA interference was achieved by transient transfection using 100 nM siRNA plus lipofectamine 2000 (Invitrogen) for 48 h. The lentiviral vectors LV‐NAA10, LV‐shNAA10 and LV‐NC (control) were from GenePharma. We generated OSCC cells stably silencing and overexpressing NAA10, according to a previous study.^36^

### 2.3 Western blot

Protein was lysed by using RIPA lysis buffer containing 1% PMSF to extract the total cellular protein. Cell lysates were incubated on ice for 30 min and then centrifuged for 15min at 13,000 *g* to remove debris. Aliquots of proteins were boiled in 1× loading buffer for 10 min, samples containing 30 µg of total proteins were resolved by SDS‐PAGE, and proteins were transferred to PVDF membrane (Millipore Corporation). Membranes were incubated with primary antibodies overnight at 4°C and appropriate HRP‐secondary antibodies for 2 h at room temperature. Protein bands were visualized using enhanced chemiluminescence detection (SuperSignal West Femto Maximum Sensitivity Substrate; Thermo Scientific). The antibody information was listed in Table S1.

### 2.4 Gene expression microarray and qRT–PCR

RNA was extracted from cells with Trizol reagent (Invitrogen). Gene expression profiles in NAA10‐silenced CAL 27 and control cells were examined by using Affymetrix‐Gene Chip Human Exon 1.0 ST arrays containing 41 000 transcripts and variants microarray, and data were deposited in Gene Expression Omnibus databank (http://www.ncbi.nlm.nih.gov/geo/query/acc.cgi?acc=GSE52723). The fold change expression was calculated after normalization. *p* value < 0.05 and the fold change threshold ≥2 were chosen to identify the statistically significant alterations. Real‐time RT‐PCR (qRT‐PCR) assays were performed following reported procedures.

### 2.5 Transwell migration and invasion assays

We performed cell migration and invasion assays according to a previous study.^19^ Briefly, 8 × 10^5^ cells were respectively plated in an uncoated top chamber (Pore size, 8 μm; Corning) for migration assay, and in a Matrigel (BD Biosciences)‐coated top chamber for invasion assay. In both assays, cells were incubated in serum‐free medium, and medium supplemented with serum was used for a chemoattractant in the lower chamber. After 24 h of incubation, the migrating cells on the lower surface of the membrane were fixed with methanol and stained with crystal violet. The number of cells through the membrane was counted under a light microscope (100×, three random fields per well).

### 2.6 In vivo tumorigenesis in nude mice

For nude mice tumorigenicity assays, 5 × 10^6^ indicated cells were injected into 6‐week‐old BALB/c female nude mice. The length (L) and width (W) of each tumour mass were measured by callipers once a week. Tumour volume was calculated according to the formula: Tumour volume = (L × W^2^)/2. After the nude mice were sacrificed, the tumours were removed and weighed, then fixed in 10% neutral buffered formalin for 24 h, embedded in paraffin, sectioned at 6‐μm thickness, and for immunohistochemical staining. The animal study was approved by the biomedical ethical committee of First Affiliated Hospital of the Medical College, Shihezi University and performed along with established National Institutes of Health guide for the care and use concordant with the United States guidelines.

### 2.7 GST‐Pull Down assays and Immunoprecipitation

Human full‐length NAA10 cDNA was cloned into pGEX‐5X‐3vector, and the recombinant Glutathione‐S‐transferases (GST)‐NAA10 protein was expressed in *Escherichia coli* and purified with Glutathione Sepharose 4B beads. 2 μg of His‐RelA/p65 (ProteinTech Group) was incubated with 2 μg of either GST‐NAA10 or GST plus glutathione beads in binding buffer at 4°C overnight, followed by washing with the same buffer for three times. Proteins were boiled in SDS loading buffer and subjected to Western blot with anti‐His and anti‐GST antibodies. For immunoprecipitation, CAL 27 and SCC‐15 cells were lysed in Cell lysis buffer (BL509A, Biosharp). Lysates (1 mg total protein) were incubated with anti‐RelA/p65 antibody (2 μg) for 16 h at 4°C followed by a 2 h incubation with 20μl immobilized Protein A/G Sepharose beads (sc‐2003), with normal mouse IgG as a negative control. The beads were washed with lysis buffer five times, and the immunoprecipitates were examined by Western blot using anti‐RelA/p65 and anti‐NAA10 antibodies.

### 2.8 Subcellular fractionation

CAL 27 and SCC‐15 Cells in the logarithmic growth period were harvested and the fractionation of nuclear and cytoplasmic proteins was performed as described previously,^10^ and the qualities of cytoplasmic and nuclear extracts were, respectively, verified by Western blot with antibodies against β‐actin and Histone H_3_.

### 2.9 Immunofluorescence

CAL 27 and SCC‐15 cells were grown on coverslips and fixed in 4% paraformaldehyde for 30 min at 4°C, followed by permeabilization with 0.5% Triton X‐100 in phosphate‐buffered saline (PBS) for 5 min and blocked with 3% bovine serum albumin at room temperature for 1 h. Anti‐NAA10 antibody and anti‐p65 antibody were then applied to the cells overnight at 4°C, followed by washing with PBS/0.1% Triton X‐100 and probing with tetramethylrhodamine isothiocyanate‐conjugated anti‐rabbit secondary antibody and fluorescein isothiocyanate‐conjugated anti‐mouse antibody for 45 min at room temperature. After washing, cells were stained with 4’,6‐diamidino‐2‐phenylindole and mounted on 50% glycerol/PBS. A Leica SP2 confocal system (Leica Microsystems) was used to observe the localization of NAA10 and RelA/p65.

### 2.10 Luciferase reporter assay

CAL 27 cells cultured in 24‐well plates were cotransfected indicated plasmids by using lipofectamine 2000. After 48 h, the luciferase activity was measured using a dual‐luciferase reporter assay kit (E1910, Promega) according to the manufacturer's protocol. The firefly luciferase intensity was normalized based on transfection efficiency measured by Renilla luciferase activity.

### 2.11 Chromatin immunoprecipitation assay

Quantitative chromatin immunoprecipitation (qCHIP) was performed as described previously.^10^ The sequences of specific primers were listed in Table S3.

### 2.12 Gene expression microarray data analysis and statistics

The raw data files from microarray profiling were imported into the Partek Genomics Suite (v. 6.6; Partek) for analysis, and two‐way analysis of variance (2‐way ANOVA) was applied with a fold change of 1.5 for the selection of differentially expressed genes at a significance level of *p* < 0.05. The differentially expressed gene lists were further correlated for their relevant biological function and reaction pathway by analysing the GSEA (Gene Set Enrichment Analysis) and KEGG (Kyoto Encyclopedia of Genes and Genomes) using the Partek Genomic Suite. A significance level of *p* < 0.05 in the GSEA analysis to identify the significant biological process involved was observed, whereas an enrichment score of *p* < 0.05 in the KEGG pathway to identify the significant pathway was observed.

### 2.13 Statistical analysis

All data for each group derived from three independent experiments were presented as the means ± SD. Statistically significant differences using a Student's *t*‐test method that was evaluated using SPSS 20.0 (SPSS). A *p* values < 0.05 was considered statistically significant.

## 3 RESULTS

### 3.1 NAA10 has tumour‐suppressive function in vitro and in vivo

**FIGURE 1:**
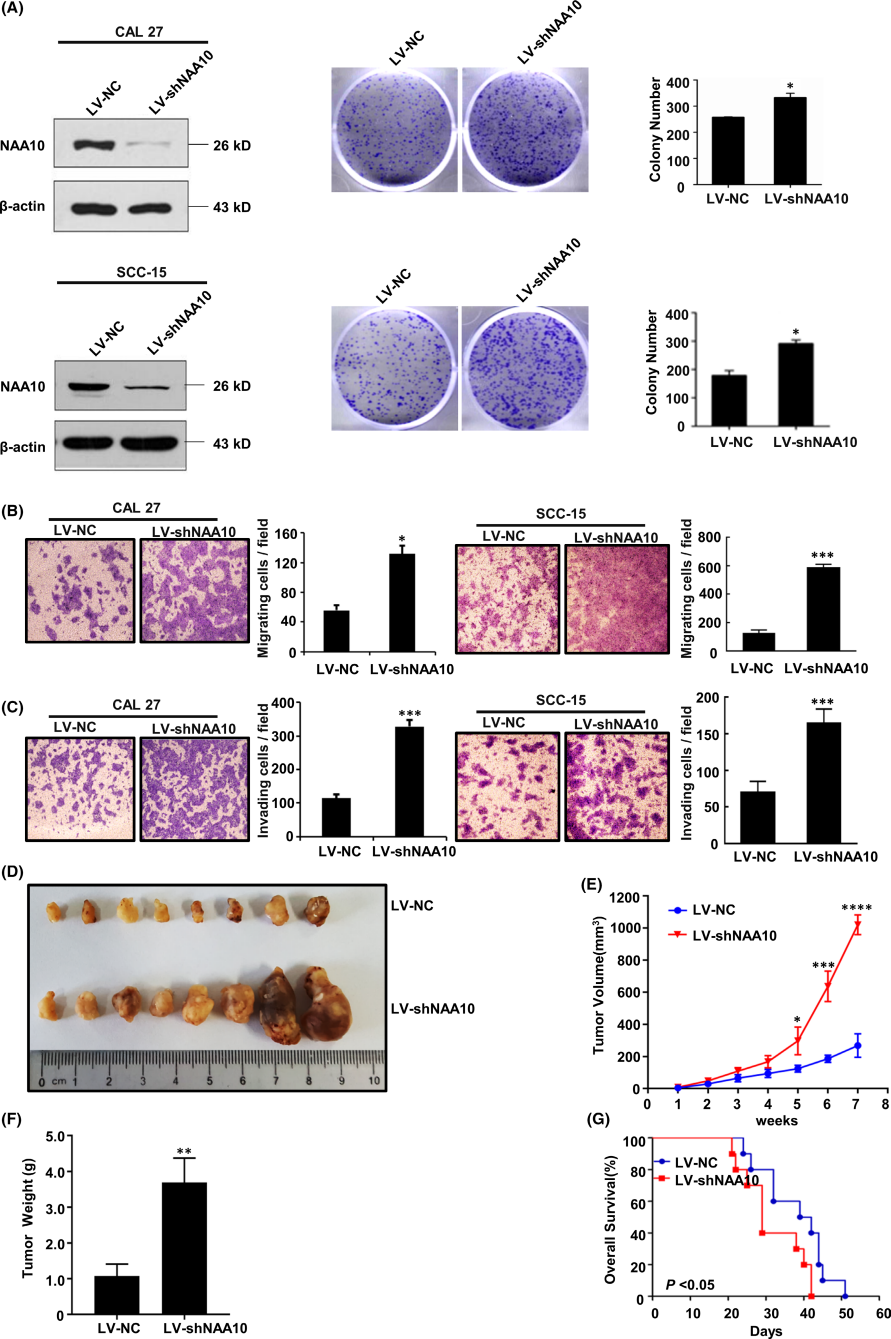
NAA10 knockdown promotes OSCC cell migration and invasion, and tumorigenesis in vivo. (A) Stable knockdown of NAA10 was examined by Western blot in CAL 27 and SCC‐15 cells, and proliferation in CAL 27 and SCC‐15 cells after Silencing of NAA10 was assessed by colony forming assay. Colonies were stained with crystal violet 10 days after seeding. (B and C) Transwell assay were performed to evaluate the effect of NAA10 silencing on migration (B) and invasion (C) of CAL 27 and SCC‐15 cells. Collective results from three independent experiments (triplicate wells for each) were summarized in the graphs. The fold difference represents the mean of triplicate experiments compared with control cells. (D–F) Macrograph, volume and weight of tumours in both two groups. Eight mice were analysed in each group. (G) Percentage survival of nude mice inoculated with indicated cancer cells. *p < 0.05, **p < 0.01, ***p < 0.005, as determined by Student's *t*‐test

Our previous study revealed that NAA10 is overexpressed in OSCC tissues, and NAA10 expression correlates to TNM stage and lymph node status. Moreover, our data confirmed that NAA10 functions as an independent prognostic factor for OSCC patients.^35^ To further explore the role of NAA10 in OSCC cells, CAL 27 and SCC‐15 cells were transfected with three siRNAs targeting NAA10, and the NAA10 expression had a better‐reduced trend in OSCC cells which were transfected with siNAA10‐2 in the level of mRNA and protein, and cell migration was significantly elevated in CAL 27 cells with siNAA10 compared with control cells (Figure S1). Next, CAL 27 and SCC‐15 cells with stable interference of NAA10 expression were generated by lentiviral infection of shRNA targeting NAA10, whose target was identical to that of siNAA10‐2. As illustrated in Figure 1A, silencing of NAA10 was confirmed by Western blot analysis in the OSCC cell lines (Left panel, Figure 1A). Subsequently, we found that knockdown of NAA10 significantly elevated the cell proliferation capacity (Right panel, Figure 1A), migration (Figure 1B) and invasion (Figure 1C) of CAL 27 and SCC‐15 cells.

Next, we evaluated the potential effects of NAA10 on tumorigenicity in BALB/c nude mice. Specifically, the growth rates of tumour xenografts were elevated by stable knockdown of NAA10 (Figure 1D–F). Consequently, mice inoculated with NAA10‐silenced cells had shorter survival time (Figure 1G). Taken together, these data indicated that NAA10 suppresses tumorigenesis and progression of OSCC in vitro and in vivo.

The acetyltransferase activity of NAA10 plays an essential role in some NAA10‐regulated biological events.^4^ To uncover whether the acetylation activity of NAA10 was involved in regulating cellular migration and invasion. Plasmids encoding R82A mutant NAA10, NAA10‐MT, which had lost its ability to associate with acetyl‐CoA, and, therefore, exhibited low acetyltransferase activity^37^ and wild‐type were transfected into CAL 27 and SCC‐15 cells. We found that the two constructs exhibited similar inhibitory effects on cellular migration and invasion (Figure S2B,C). Taken together, these results indicated that NAA10 regulates cell migration and invasion through a mechanism‐independent intrinsic acetyltransferase activity.

### 3.2 NAA10 knockdown inhibits P53 signalling pathway

**FIGURE 2:**
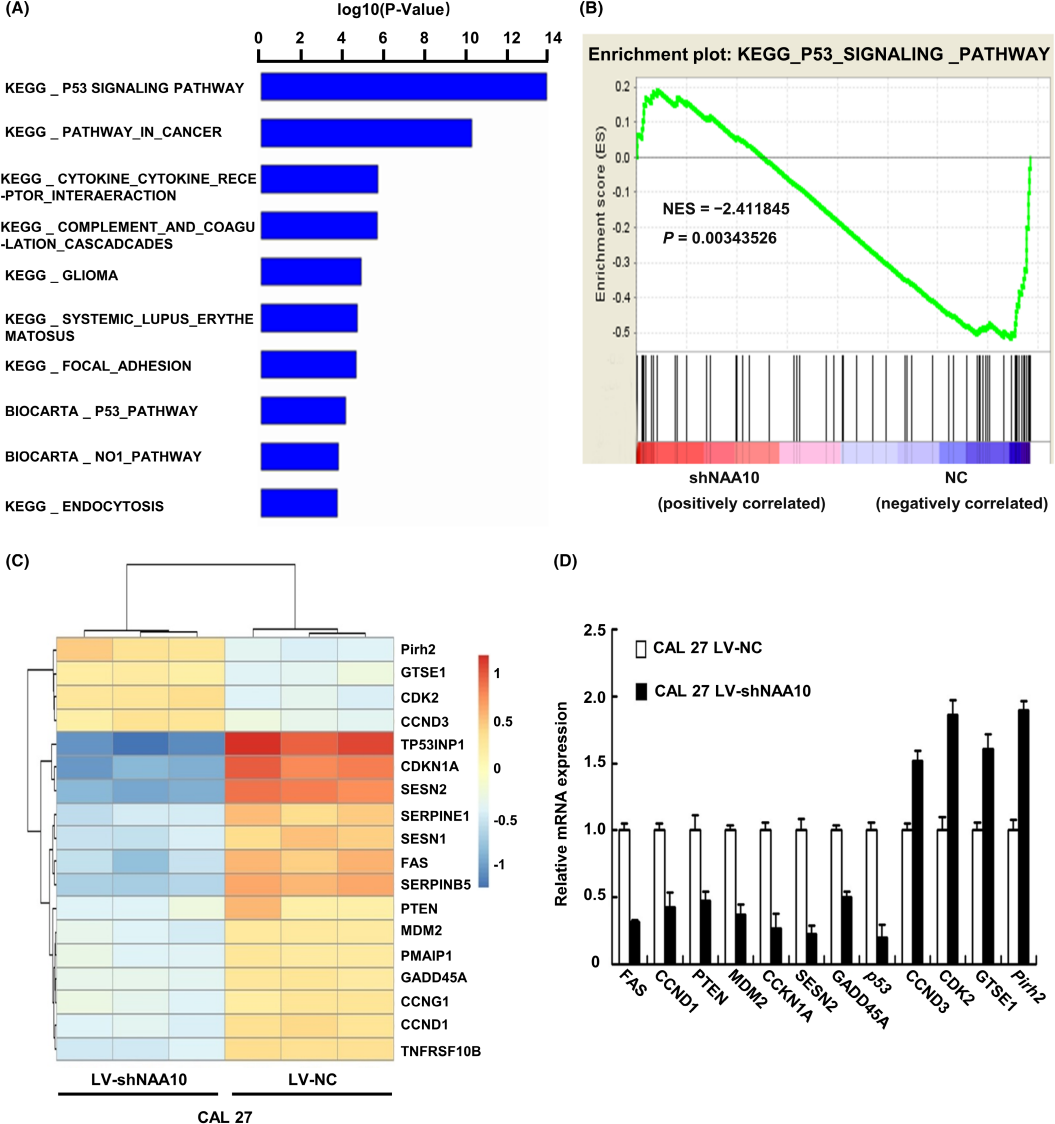
NAA10 expression level is closely associated with Pirh2‐p53 signalling pathway. (A) *NAA10* gene expression profile was analysed after the stable interference of CAL 27 cells, and genes with differential expression of more than two folds were selected for KEGG analysis. The top 10 KEGG pathways were displayed. (B) GSEA result showed that high NAA10 expression was positively correlated with p53 signalling in OSCC. NES, normalized enrichment score. (C) Differentially expressed genes of P53 signalling affected by NAA10 silencing in CAL 27 cells. The colour intensity was proportional to the log_2_ of expression ratio (blue, downregulated; red, upregulated). (D) Expression levels of selected genes in P53 pathway were examined by qRT‐PCR in CAL 27 cells. Each bar represents the mean ± SD from three independent experiments

Next, we sought to gain insight into the mechanism by which NAA10 regulates invasion and metastasis phenotype in OSCC. We performed cellular Gene Expression Profile with NAA10 stably silenced CAL 27 cells, and the differentially regulated genes were selected for KEGG pathway analysis. The analysis result showed that the P53 signalling pathway was the most relevant downstream signalling pathway of NAA10 (Figure 2A). Next, we performed Gene Set Enrichment Analysis (GSEA) and found that P53 signalling pathway was enriched in this dataset (Figure 2B). Subsequently, we performed the genetic variations of P53 signalling pathways of clustering analysis (Figure 2C). Consequently, some genes in P53 signalling pathway were determined by qRT–PCR, and *Pirh2* was upregulated and *p53* downregulated after knocking down NAA10 (Figure 2D). p53 is a major substrate of Pirh2, and Pirh2 promotes p53 degradation.^32^ Furthermore, we previously demonstrated that the expression of NAA10 was negatively correlated with that of Pirh2 in OSCC.^35^ Therefore, the Pirh2‐p53 signalling pathway is selected for verification and study in the next step.

### 3.3 NAA10 attenuates MMPs expression via the Pirh2‐p53 signalling pathway

**FIGURE 3:**
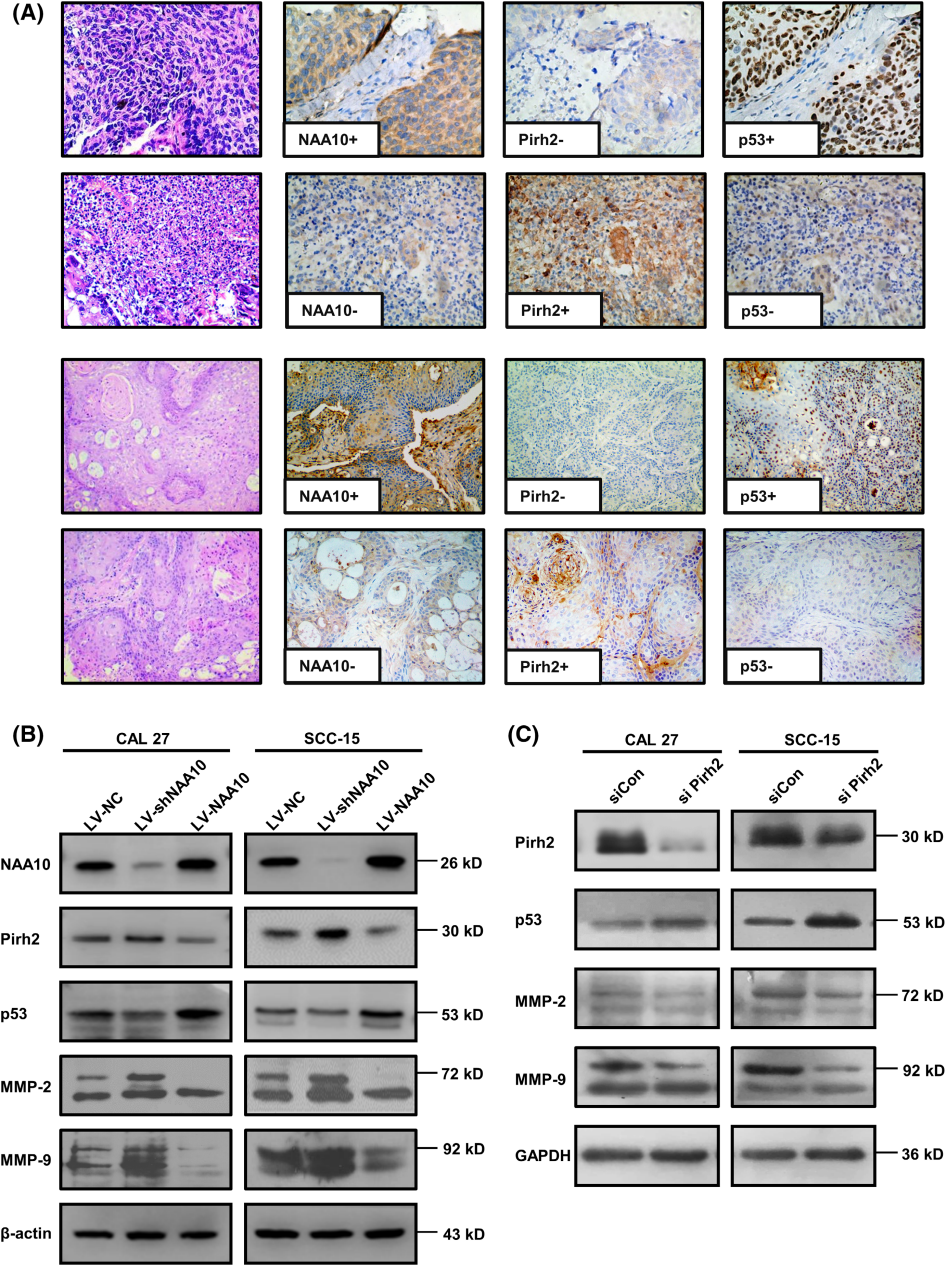
NAA10 regulates the expression of related proteins involved in the Pirh2‐p53 signalling pathway. (A) The left‐most column: HE staining images in human OSCC tissue microarrays (TMAs) and tumour xenograft tissues of nude mice. The rest of three columns: Immunostaining of NAA10, Pirh2, p53 in human OSCC tissue microarrays (TMAs) and tumour xenograft tissues of nude mice. Magnification, 100×. (B) Stable silencing of endogenous NAA10 increased expression of Pirh2, MMP‐2 and MMP‐9, but decreased p53. And stable expression of ectopic NAA10 decreased expression of Pirh2, MMP‐2 and MMP‐9, but increased p53 in indicated cells. (C) Endogenous Pirh2 expression in CAL 27 and SCC‐15 cells was silenced by siRNA Scrambled siRNA (siCon) was used as a negative control. The effect of silencing Pirh2 on p53, MMP‐2 and MMP‐9 expression was detected by Western blot. The results were performed in triplicate

To uncover whether NAA10 was involved in regulating P53 signalling pathway. Firstly, human OSCC tissue microarrays (TMAs) and tumour xenograft tissues of nude mice were performed HE staining and staining images were viewed and evaluated independently by two experienced pathologists to ensure that the selected tissues were squamous cell carcinoma. Next, we verified the expression correlation of NAA10, Pirh2 and p53 by immunohistochemical staining, which suggested that NAA10 abundance was negatively associated with that of Pirh2 but positively associated with that of p53 (Figure 3A). Moreover, tumour invasion is often associated with the enhanced synthesis of matrix metalloproteinases (MMPs), among which MMP‐2 and MMP‐9 are of central importance.^38^ Thus, we sought to determine the expression level of Pirh2, p53, MMP‐2 and MMP‐9 protein in CAL 27 and SCC‐15 cells after silencing NAA10. Western blot showed that NAA10 stable knockdown significantly increased Pirh2, MMP‐2 and MMP‐9 expression, and decreased p53 expression. Consistently, NAA10 stable overexpression inhibited the level of Pirh2, MMP‐2 and MMP‐9, and elevated p53 expression (Figure 3B).

To determine whether Pirh2 inhibited the expression of p53 and modulated p53‐dependent expression of MMPs in OSCC cells, the expression of Pirh2 in OSCC cells was silenced by RNA interference (RNAi). The result showed that Pirh2 knockdown could dramatically increase p53 and decrease MMP‐2, MMP‐9 in CAL 27 and SCC‐15 cells (Figure 3C). These results emphasized the important role of NAA10 in the P53 pathway and suggested that NAA10 induces Pirh2 reduction and rescues p53 expression.

### 3.4 NAA10 interacts with RelA/p65 and attenuates phosphorylation of p65 in OSCC

**FIGURE 4:**
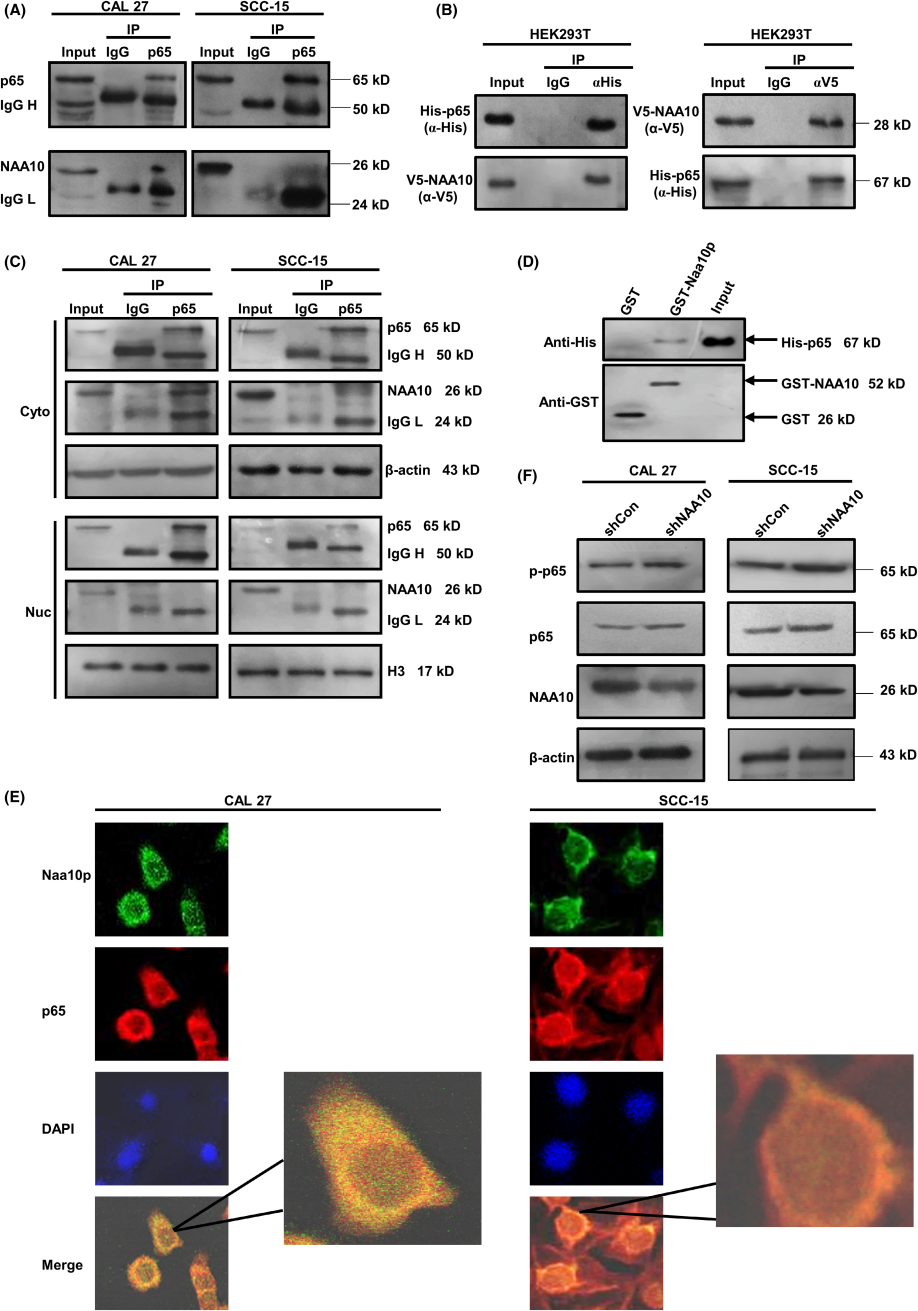
NAA10 is associated with RelA/p65 and influences the phosphorylation of p65. (A) Immunoprecipitation of NAA10 and RelA/p65 with an anti‐RelA/p65 antibody in CAL 27 and SCC‐15 cells. IgG H, IgG heavy chain; IgG L, IgG light chain. (B) Protein interaction of NAA10 with RelA/p65 proteins in vitro. V5–NAA10 and His–p65 were cotransfected in 293T cells, and reciprocal immunoprecipitation and immunoblotting were carried out with the indicated antibodies. (C) Cytoplasmic and nuclear interactions between NAA10 and RelA/p65. Indicated cells were subjected to subcellular fractionation, and the qualities of cytoplasmic and nuclear extracts were, respectively, verified by Western blot with antibodies against β‐actin and H_3_. Cytoplasmic protein (500 µg) and nuclear protein (200 µg) were immunoprecipitated with an anti‐p65 antibody, followed by Western blot with anti‐NAA10 and anti‐RelA/p65 antibodies. (D) Binding assay of His‐p65 with GST or GST‐NAA10. (E) Colocalization of NAA10 and RelA/p65. CAL 27 and SCC‐15 cells were subjected to immunofluorescence staining with anti‐NAA10 (green) and anti‐RelA/p65 (red) antibodies. Colocalization was shown by the merge (yellow). Nuclei were counterstained with 4’,6‐diamidino‐2‐phenylindole (DAPI) (blue). (F) shCon and shNAA10 were transfected into CAL 27 and SCC‐15 cells, respectively. The endogenous level of p65 and p‐p65 (Ser536) was detected by Western blot. All experiments were carried out in triplicate

In the previous study, there was a significant inverse correlation between the expression of NAA10 and Pirh2 in OSCC patient tissues.^35^ Furthermore, the effect of NAA10 on the expression of Pirh2 was determined by qRT‐PCR in OSCC cells. The results indicated that *NAA10* down‐regulates the mRNA expression of *Pirh2* (Figure S3). These data raised a possibility that NAA10 regulates Pirh2 expression at the transcriptional level. Accumulating evidence demonstrated that NAA10 interacts with various transcription factors to regulate the expression of tumour‐related target genes.^10^, ^11^, ^13^ Firstly, we selected the promoter region of Pirh2 and scanned the proximal 1907bp of the promoter region of Pirh2 using Promo software (http://alggen.lsi.upc.es/cgi‐bin/promo_v3/promo/ promoi nit.cgi?dirDB=TF_8.3) and identified potential transcription factors (TF). Interestingly, we scanned the binding sites of these TF in the promoter region of Pirh2 of proximal 1907bp using Jaspar database (http://jaspardev.genereg.net/) and identified RelA/p65 with high score and more binding sites in Pirh2 promoter. Thus, we hypothesized whether RelA/p65 has a transcriptional activation effect on Pirh2, and NAA10 suppresses RelA/p65‐mediated transcription activity of Pirh2 by interacting with the RelA/p65. Immunoprecipitation assays were performed to uncover the interaction between NAA10 with RelA/p65, and the results of immunoprecipitation analyses of endogenous or exogenous proteins of NAA10 and RelA/p65 in 293T and OSCC cells revealed that NAA10 physically interacted with RelA/p65 in vivo and in vitro (Figure 4A,B). To further investigate the subcellular interaction of NAA10 and RelA/p65, CAL 27 and SCC‐15 cells were fractionated to acquire cytoplasmic and nuclear proteins. Next, we performed immunoprecipitation assay with cytoplasmic and nuclear proteins and demonstrated the presence of physical interaction was mainly in the cytoplasm (Figure 4C). In vitro GST‐Pull Down assay confirmed the association between GST‐tagged NAA10 and His‐RelA/p65, indicating a direct binding between the two molecules (Figure 4D). By immunofluorescence staining and confocal microscopic observation, colocalization of NAA10 and p65 in CAL 27 and SCC‐15 cells was revealed mainly in the cytoplasm (Figure 4E).

To determine the effect of NAA10 on the expression of p65 and p65 phosphorylation. NAA10 was knockdown in CAL 27 and SCC‐15 cells through shRNA transfection. The Western blot results showed that the expression p‐p65 (serine‐536) was significantly elevated through shRNA transfection, while p65 did not change (Figure 4F). And, densitometric data showed a considerably higher ratio of p‐p65 to p65 in OSCC cells with knockdown of NAA10 in comparison with control cells (*p* < 0.01, Figure S4).

### 3.5 NAA10 suppresses RelA/p65‐activated Pirh2 transcription

**FIGURE 5:**
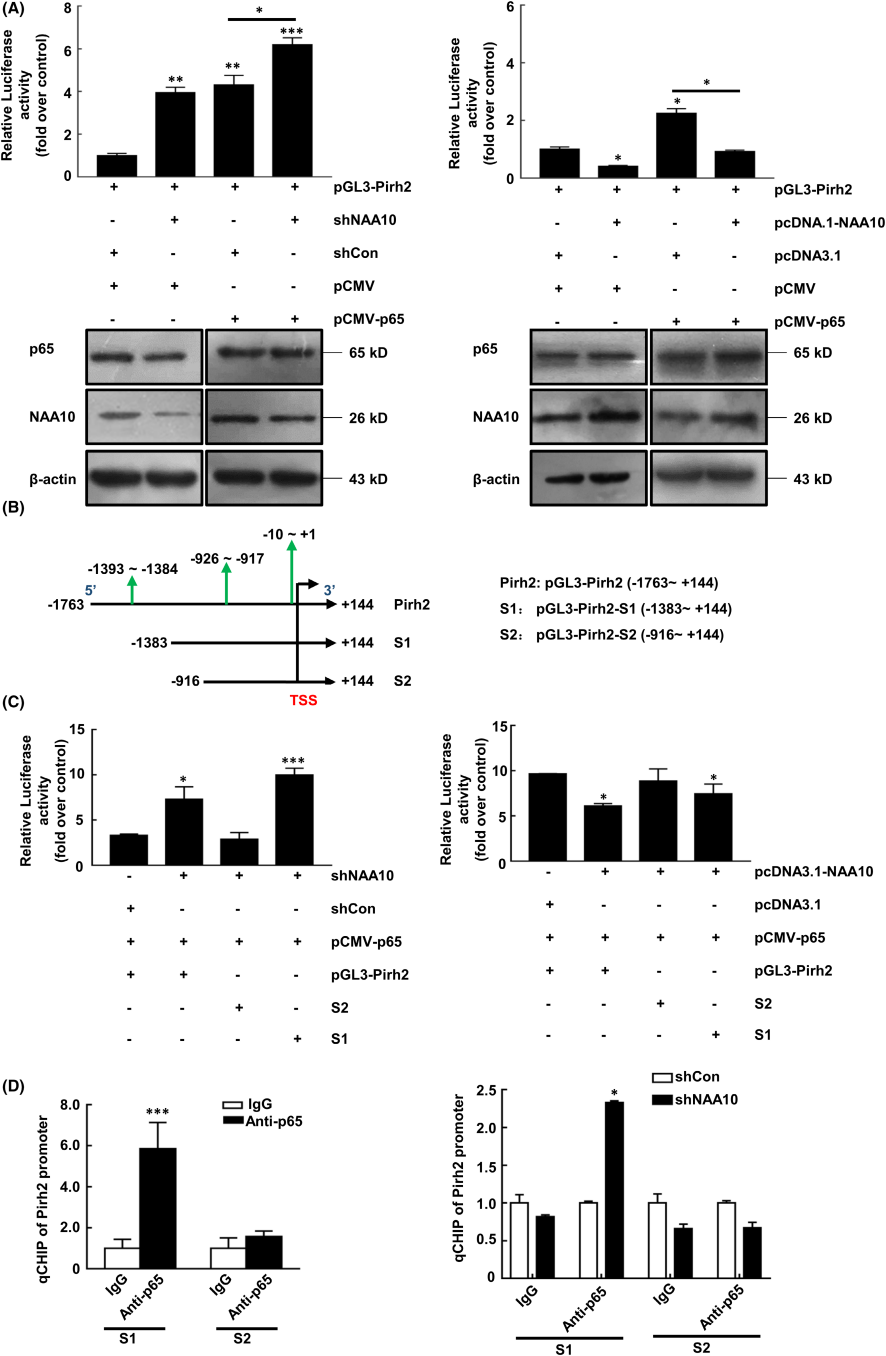
NAA10 inhibits RelA/p65‐activated transcriptional expression of Pirh2. (A) NAA10 cooperated with RelA/p65 to regulate Pirh2 expression in luciferase assay. (B) According to transcription factor RelA/p65 binding sites in the Pirh2 promoter: site 1 (Pirh2p1), −1393 to −1384; and site 2 (Pirh2p2), −926 to −917; and site 3 (Pirh2p3), −10 to +1. Truncated Pirh2 promoter constructs were established. (C) Truncated Pirh2 promoter constructs were transfected to CAL 27 cells, respectively, and relative luciferase activities were determined. (D) CHIP‐qPCR assay demonstrated the direct binding of RelA/p65 to the Pirh2 promoter. Collective results were carried out in triplicate. The bars represent the mean ± SD of three independent experiments. *p < 0.05, ***p < 0.005

Our results raised the possibility that NAA10 could modulate the signalling events upstream of Pirh2 transcription, thereby promoting Pirh2 mRNA expression. Thus, we speculated whether RelA/p65 has a transcriptional activation effect on Pirh2, and NAA10 suppresses RelA/p65‐mediated transcription activity of Pirh2 by interacting with the RelA/p65. Next, we explored whether RelA/p65 can affect the transcription of Pirh2, the human Pirh2 promoter‐luciferase plasmid pGL3‐Pirh2 and the RelA/p65 overexpression plasmid were cotransfected to CAL 27 cells, and the transcriptional activation was detected. The luciferase activity was significantly higher than in the control group after RelA/p65 overexpression (Lanes 1 and 3 in Left and Right panels; Figure 5A). The result elucidated that RelA/p65 has a transcriptional activation effect on Pirh2. Furthermore, we showed that overexpression of RelA/p65 augmented Pirh2’s promoter activity, which was enhanced by silencing of NAA10 but was compromised by co‐expression of NAA10 (Lane 3 and 4 in Left and Right panels; Figure 5A), indicating NAA10 could alleviate RelA/p65‐regulated Pirh2 transcription.

To elucidate the RelA/p65 and Pirh2 promoter‐specific binding sites, the truncated luciferase reporter plasmid: S1: pGL3‐Pirh2‐S1 (−1383 ~ +144); S2: pGL3‐Pirh2‐S2 (−916 ~ +144) of human Pirh2 promoter (length 1907 bp) was further constructed based on identified three potential RelA/p65 binding sites on the promoter region of Pirh2 by using Promo software (Figure 5B). Then, the truncated luciferase reporter plasmids were cotransfected to CAL 27 cells with p65 overexpression plasmid and NAA10 knockdown or overexpression plasmid, respectively. The results showed that the luciferase activity of the truncated Pirh2 promoter region S1 was increased after NAA10 knockdown and is comparable with the full‐length Pirh2 promoter. However, the luciferase activity of the truncated S2 had no significant difference with that of the control group (Figure 5C). Similar data could be observed following stable expression of NAA10 (Right panel, Figure 5C).

We performed chromatin immunoprecipitation (ChIP)‐qPCR assays in CAL 27 cells, and the results revealed that p65 bound to S1 site, but not to S2 site in qCHIP assays (Left panel, Figure 5D). Once NAA10 was knockdown, binding of RelA/p65 to S1 site was dramatically increased (Right panel, Figure 5D), suggesting that binding of RelA/p65 to S1 site was negatively regulated by the cellular levels of NAA10. Collectively, these data indicated that RelA/p65 binds to the promoter region S1 to regulate Pirh2 transcription and NAA10 suppresses RelA/p65 induced Pirh2 transcription.

### 3.6 Pirh‐p53 signalling pathway is essential for NAA10 medicated OSCC tumour suppressor function

**FIGURE 6:**
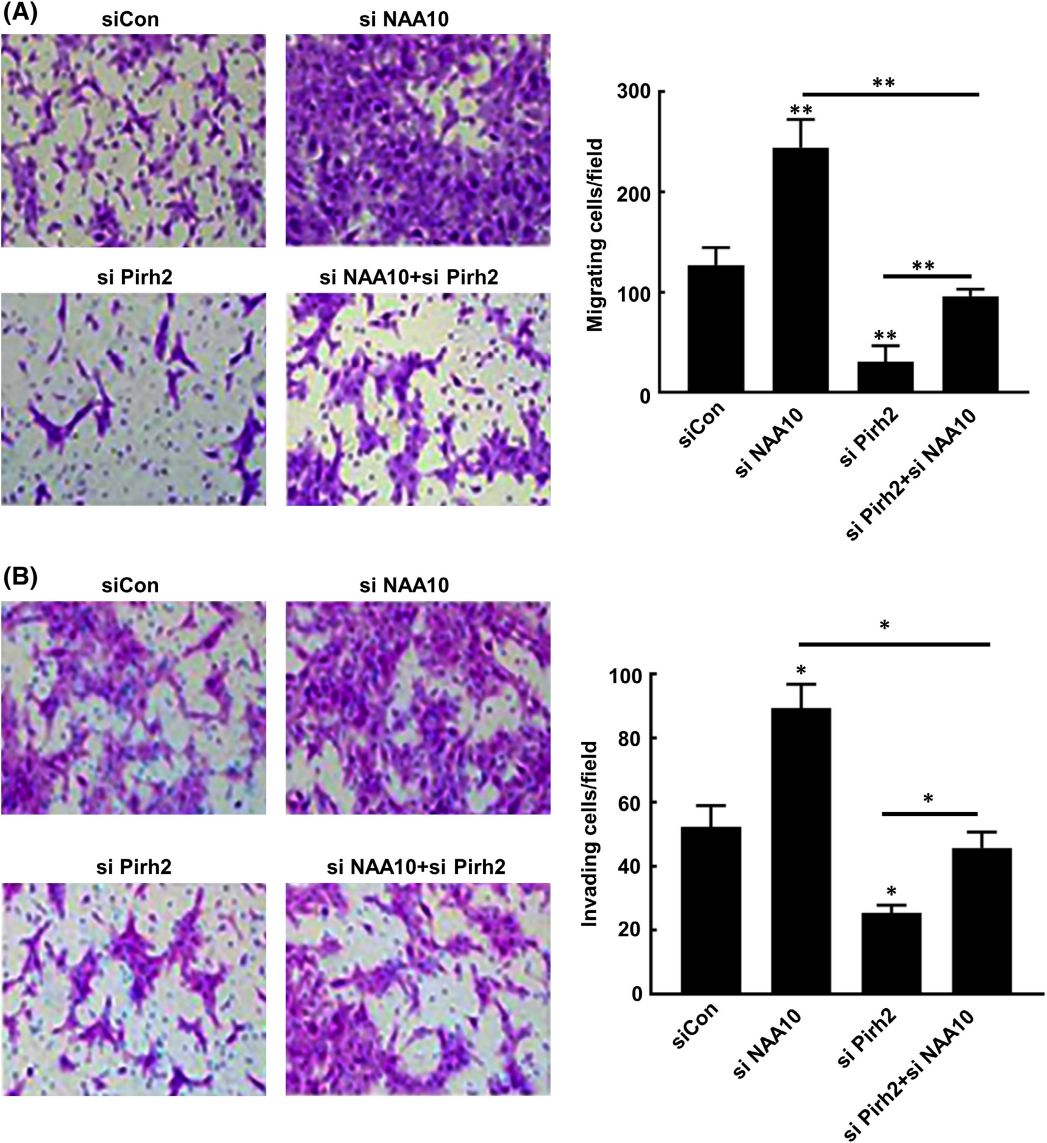
Pirh2‐p53 signalling pathway is essential for NAA10 medicated OSCC invasion and metastasis. (A and B) Transwell assays were used to evaluate whether Pirh2 knockdown blocked the promoting effects of silencing NAA10 on the migration (A) and invasion (B) of CAL 27 cells. Values are presented as the means ± SD of three independent experiments. *p < 0.05, **p < 0.01

To further confirm that NAA10 affects the migration and invasion of OSCC through Pirh2‐p53 signalling pathway, CAL 27 cells were transfected with *NAA10* siRNA or *Pirh2* siRNA, respectively, or in combination. Notably, silencing of NAA10 elevated cellular migration and invasion, and while silencing of Pirh2‐inhibited cellular migration, invasion in CAL 27 cell. Pirh2 silencing indeed relieved the cell migration induced by NAA10 knockdown (Figure 6A). Similar results were also observed in cell invasion (Figure 6B). These data suggested that NAA10 plays a role in the migration and invasion of OSCC cells in part via regulating Pirh2‐p53 signalling pathway.

## 4 DISCUSSION

In this study, we demonstrated the interaction between RelA/p65 subunit of NF‐κB with NAA10, and NAA10 suppressed Pirh2 expression via RelA/p65‐dependent transcription in OSCC cells. Pirh2 is a Ring‐H2 domain containing E3 ubiquitin ligase, which targets several suppressors genes including p53.^32^ Emerging evidence further confirmed that Pirh2 might be a novel critical oncoprotein in the development and progression of tumour. Overexpression of Pirh2 had been found in various cancers, including head and neck squamous cell carcinoma (HNSCC). And, Pirh2 expression was correlated with poor prognosis, at least partially through degradation of p27 in HNSCC.^33^

The emerging evidence demonstrated that p53 plays an important role in inhibiting tumour invasion and metastasis by regulating the expression and activity of Matrix metalloproteinases (MMPs).^24^, ^25^, ^28^, ^31^ Recently, Lee et al. found that p53 is implicated in the regulation of EMT process, and p53 inhibits the expression of E‐cadherin and increases that of Snail in OSCC cells.^39^ Moreover, p53 inhibited the invasion of OSCC cells via the suppression of MMP‐13 expression.^31^ However, p53 has been shown to be ubiquitylated by Pirh2 in vitro and in vivo, and overexpression of Pirh2 decreased p53 levels and suppressed p53‐dependent transactivation and biological function.^34^ Indeed, the overexpression of Pirh2 was concomitant with decreased p53 levels in malignant tissues, suggesting a role for Pirh2 in tumorigenesis through regulation of p53 stability. Abrogation of Pirh2 results in a steady increase in p53 cellular levels.^34^ Consistently, we found that the enhancement of p53 inhibited the expression of MMP‐2 and MMP‐9 in CAL 27 and SCC‐15 cells after Pirh2 silencing (Figure 3C). These data provide evidence, Pirh2 promotes tumour invasion and metastasis through suppressing p53.

**FIGURE 7:**
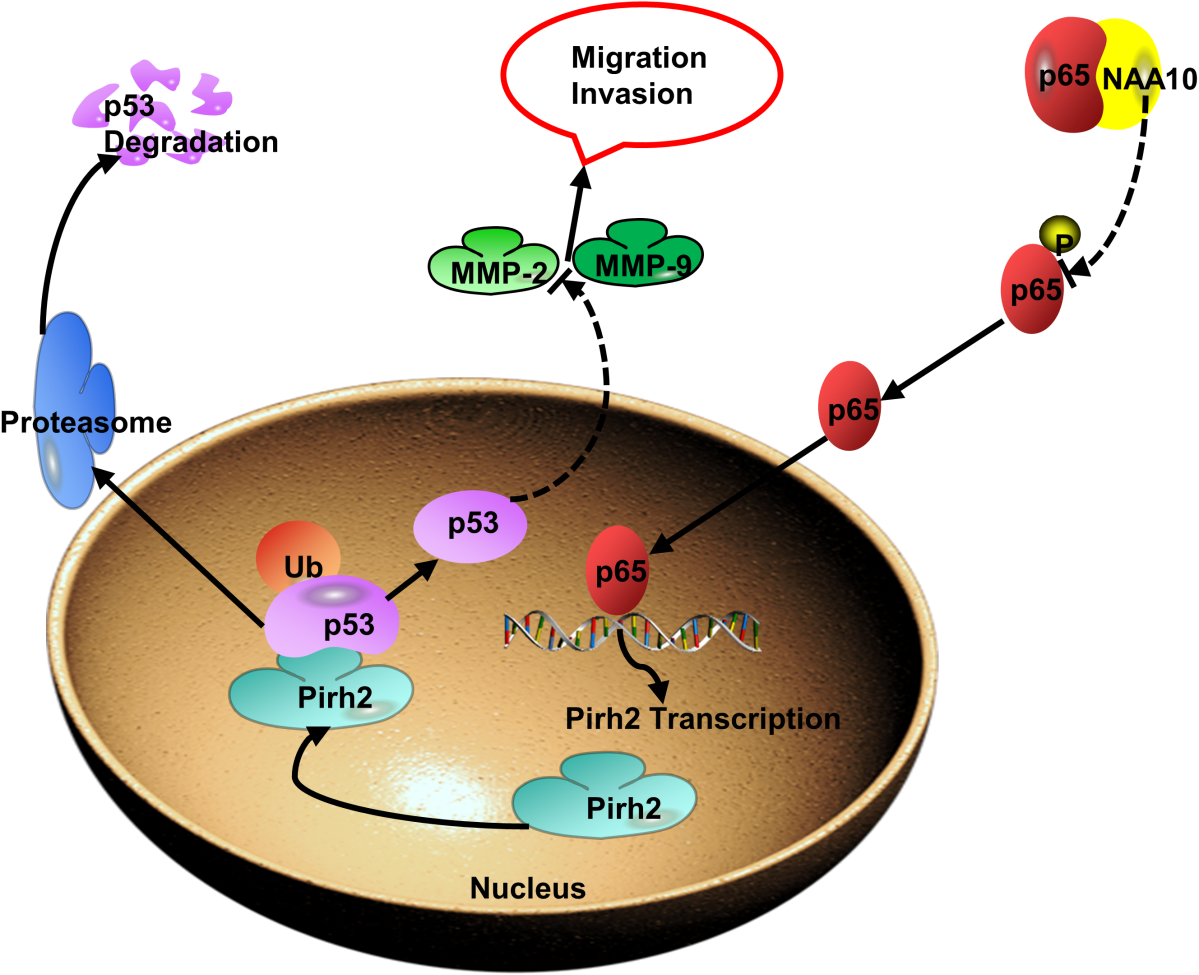
Schematic representation depicting the effects of NAA10 on OSCC invasion and metastasis. NAA10, by interacting with p65 and inhibiting the phosphorylation of p65, inhibits p65 goes into the cell nucleus and binds to Pirh2 promoter, and further attenuates the transcription expression of Pirh2 gene. Thereby the inhibition of Pirh2 increased the expression of the down‐stream tumour suppressor genes p53, which leads to the down‐regulation of the expression of the downstream MMP‐2 and MMP‐9 genes and plays the role of inhibiting the invasion and metastasis of OSCC

In the present study, we found that NAA10 inhibits the migration, invasion of OSCC cells, and attenuates xenograft tumorigenesis in vivo (Figure 1). Mechanistically, NAA10 decreases Pirh2 expression, and thus, it rescues p53 expression and decreases the expression of MMP‐2 and MMP‐9 to block migration and invasion of OSCC cells (Figure 7). We previously found that the expression of NAA10 was negatively correlated with that of Pirh2 in OSCC tissues. Besides, positive NAA10 and negative Pirh2 might be independent biomarkers for better prognosis in OSCC patients.^35^ Furthermore, the effect of *NAA10* on the expression of Pirh2 was determined by qRT‐PCR in OSCC cells. The results indicated that *NAA10* down‐regulates the mRNA expression of Pirh2 (Figure S3), suggesting a possibility that NAA10 regulates Pirh2 expression at the transcriptional level. NF‐κB is a critical transcription factor activated in various types of human cancers and plays a crucial role in tumour development and progression by inducing transcription of various target genes that modulate cell invasion, proliferation, apoptosis, survival and angiogenesis.^10^, ^40^, ^41^ We previously discovered that NAA10 interacted with RelA/p65 in colon cancer cell lines and lung cancer cell lines^10^ and now we further found that NAA10 interacts with RelA/p65 mainly in the cytoplasm and attenuates phosphorylation of p65 (Serine‐536) in OSCC cells (Figure 4). Interestingly, p65 phosphorylated on serine536 translocated to the nucleus following activation, and this nuclear translocation is not regulated by IκBα.^42^ Here, we hypothesized that NAA10 interacts with p65 in the cytoplasm to inhibit p65 translocation from cytoplasm into the nucleus, resulting in suppressed activation of NF‐κB, and thereby inhibiting Pirh2 transcription in OSCC cells. However, our results did not elucidate the mechanism that NAA10 regulates the phosphorylation of p65. Phosphorylation of p65 is an important active form, which is regulated by various kinases and phosphatases. Activation of IKK kinase leads to phosphorylation of IκBα, which is separated from the p65‐p50 complex, and then p65 enters the nucleus and functions as a transcription factor.^43^ Whether the inhibition of p65 phosphorylation by NAA10 in OSCC cells is dependent on the IKKs deserves further studies.

Based on the above results, we speculated whether RelA/p65 has a transcriptional activation effect on Pirh2, and NAA10 suppresses RelA/p65‐mediated transcription activity of Pirh2. Therefore, we constructed the luciferase plasmid of the Pirh2 promoter region and detected the transcriptional activation of Pirh2 by RelA/p65 by using luciferase reporter assay. The results demonstrated that RelA/p65 binds to Pirh2 promoter to regulate its transcription, and NAA10 suppresses RelA/p65 binding to Pirh2 promoter, thus inhibiting Pirh2 expression. Hua et al.’s study reported NAA10 decreases GIT‐assisted localization of PIX on membrane protrusions, thus alleviating CDC42/ RAC1‐dependent cell metastasis.^19^ In addition, Lee et al.^21^ found silencing of NAA10 resulted in diminished recruitment of DNMT1 to E‐cadherin promoter in qCHIP assay, but silencing DNMT1 had no effects on NAA10’s binding to the same site. They also showed that NAA10 could stabilize DNMT1–DNA association by interaction with both non‐methylated and hemimethylated DNA.

Taken together, this study elucidated that NAA10, as a tumour suppressor, inhibited tumorigenesis, migration and invasion in OSCC. Mechanically, we demonstrated that NAA10‐suppressed RelA/p65 mediated the transcription of Pirh2, and decreased its expression. Therefore, NAA10 elevated p53 protein expression and stability via impairing the effect of Pirh2 on p53 protein degradation, and thus, inhibited p53 downstream genes expression involved in migration and invasion of tumour, such as MMP‐2 and MMP‐9 (as illustrated in Figure 7). Our study suggested that NAA10 may serve as a therapeutic target for the prevention of metastasis in OSCC.

## CONFLICT OF INTEREST

The authors declare that they have no competing interests.

## AUTHOR CONTRIBUTIONS

**Fazhan Wang:** Formal analysis (equal); Software (equal); Writing – original draft (lead). **Jun Zheng:** Investigation (equal); Resources (equal). **Jie Yang:** Data curation (equal); Formal analysis (equal). **Ting Luo:** Data curation (equal); Formal analysis (equal). **Jiang Xu:** Investigation (equal); Resources (equal). **Yongyong Yang:** Methodology (equal); Writing – review & editing (equal). **Yongqing Gu:** Conceptualization (equal); Methodology (equal); Writing – review & editing (equal). **Yan Zeng:** Funding acquisition (lead); Writing – review & editing (equal).

## Supporting information

Supplementary Material

## Data Availability

All data generated or analysed during this study are included in this published paper.
