# Supplementary material for: N‐α‐Acetyltransferase 10 inhibits invasion and metastasis of oral squamous cell carcinoma via regulating Pirh2‐p53 signalling pathway
Source: J Cell Mol Med. 2022 Apr 2;26(10):2921–34. doi: 10.1111/jcmm.17306 (PMC9097830; doi:10.1111/jcmm.17306)
Supplement: Supplementary file 1 — Supplementary Material [file JCMM-26-2921-s001.doc]

**Additional file**

**Table S1. Antibodies used in this study.**

| **Antibody** | **Catalogue No** | **Source** | **Manufacturer** | **Working Concentrations** |
| --- | --- | --- | --- | --- |
| His-tag | AF5060 | Mouse, IgG | Beyotime | WB: 1:1000 |
| GST-tag (1B9) | MB8021 | Mouse, IgG | Bioworld | WB: 1:5000 |
| V5-tag | 14-6796-80 | Mouse, IgG2b | Invitrogen | WB: 1:1000 |
| Histone H3 | #4499 | Rabbit, IgG | CST | WB: 1:2000 |
| Phospho p65 (Ser536) | #3033 | Rabbit, IgG | CST | WB: 1:1000 |
| NFκB p65 | 10745-1-AP | Rabbit, polyclonal | Proteintech | IF: 1:100 |
| NFκB p65 (F-6) | sc-8008 | Mouse, IgG1 | Santa Cruz | WB: 1:1000;  IP, qCHIP: 1μg/ml |
| MMP-9 | #13667 | Rabbit, IgG | CST | WB: 1:1000 |
| MMP-2 | 10373-2-AP | Rabbit, polyclonal | Proteintech | WB: 1:500 |
| p53 (DO-1) | sc-126 | Mouse, IgG2a | Santa Cruz | WB: 1:500 |
| Pirh2 | ab189907 | Rabbit, IgG | Abcam | WB: 1:1000 |
| NAA10 (A-10) | sc-373920 | Mouse, IgG2a | Santa Cruz | WB: 1:1000; IF: 1:200 |
| β-actin | TA-09 | Mouse, IgG3 | ZSGB-BIO | WB: 1:1000 |
| GAPDH | TA-08 | Mouse, IgG1 | ZSGB-BIO | WB: 1:20000 |

**Table S2. siRNAs used in this study.**

| si NAA10-1 | 5’-CGAGCCAUGAUAGAGAACU-3’ |
| --- | --- |
| si NAA10-2 | 5'-CCCUGCACCUCUAUUCCAA-3' |
| si NAA10-3 | 5’- GGAGAGCAAAGGCAAUUCA-3’ |
| si Pirh2 | 5'-CCAACAGACUUGUGAAGAA-3' |
| si Con | 5’-UUCUCCGAACGUGUCACGU-3’ |

**Table S3. Sequences of the primers used in this study.**

| **Primers** | | **Sequences (5'-3')** |
| --- | --- | --- |
| *GAPDH* | | (Forward) GCACCGTCAAGGCTGAGAAC |
|  |  | (Reverse) ATGGTGGTGAAGACGCCAGT |
| *NAA10* | | (Forward) ATGAACATCCGCAATG |
|  |  | (Reverse) ACAATCTTCCCATTCTC |
| *Pirh2* | | (Forward) AAAATTCAACATGCCCAACAGAC |
|  |  | (Reverse) GGACCAATCCTACAAATTCCACA |
| *p53* | | (Forward) GAGGTTGGCTCTGACTGTACC |
|  |  | (Reverse) TCCGTCCCAGTAGATTACCAC |
| *Pirh2*  (for ChIP) | S1 | (Forward) ACCGGAAAGCTGAAGGGGAA |
|  |  | (Reverse) TCATACTCGGGGTACACAGCT |
|  | S2 | (Forward) AGCGAAGACTGCGACTAACGT |
|  |  | (Reverse) AGAAGCTGCGCCTCTCTAGC |

**Figure S1. Knockdown of NAA10 by three siRNAs in OSCC cells.**

**
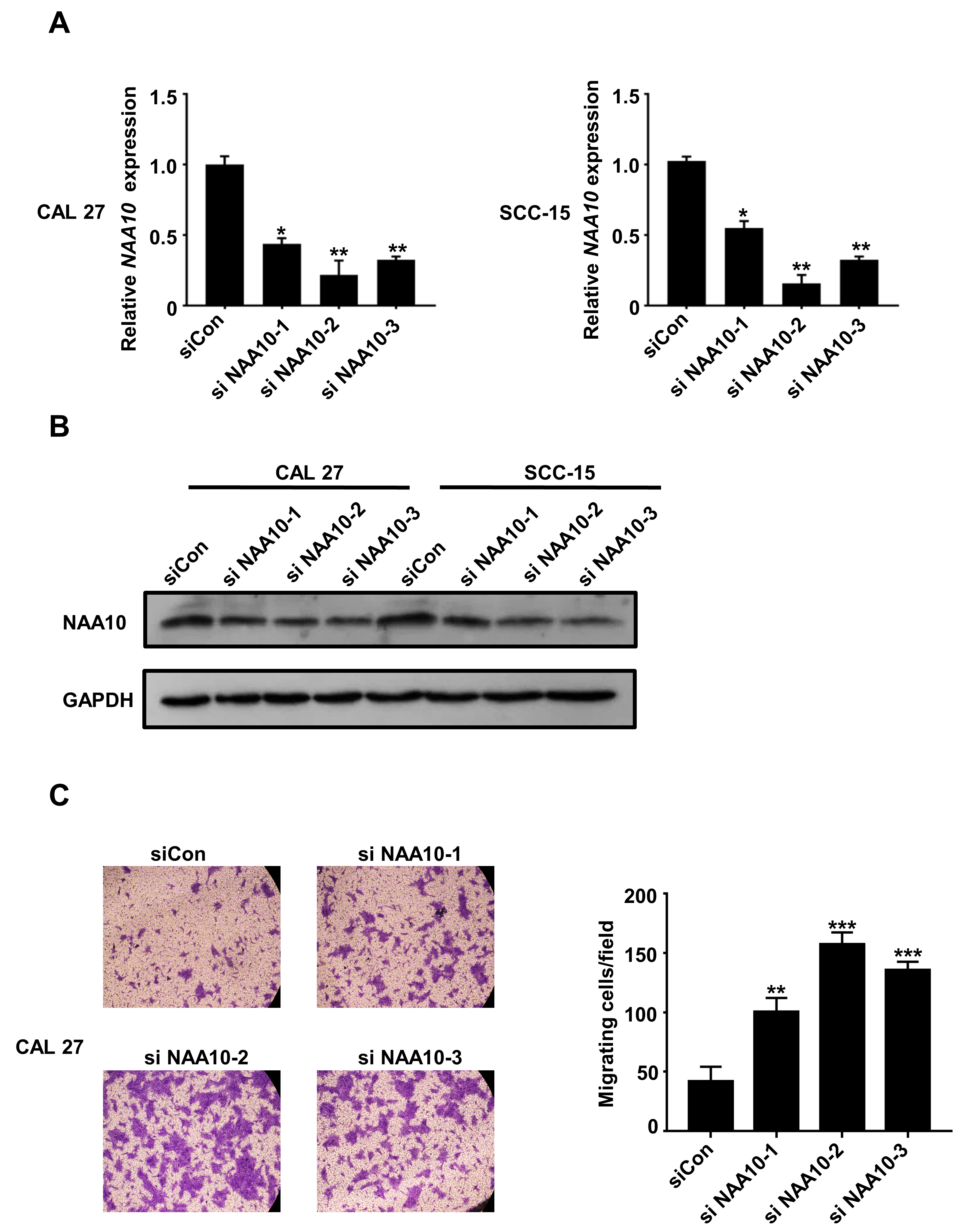
**

A, Knockdown efficiencies of three NAA10 siRNAs were determined by qRT-PCR in CAL 27 and SCC-15 cells. B, Knockdown efficiencies of three NAA10 siRNAs were determined by Western blot in CAL 27 and SCC-15 cells. C, SCC-15 cells were transfected with three different interference fragments targeting NAA10, and the effect of NAA10 knockdown on the migration of OSCC cells was detected by Transwell assay. Results were presented as the mean ± SD of three repeated experiments.

**Figure S2. NAA10’s acetyltransferase activity is not required for suppressing migration and invasion in OSCC cells.**

**
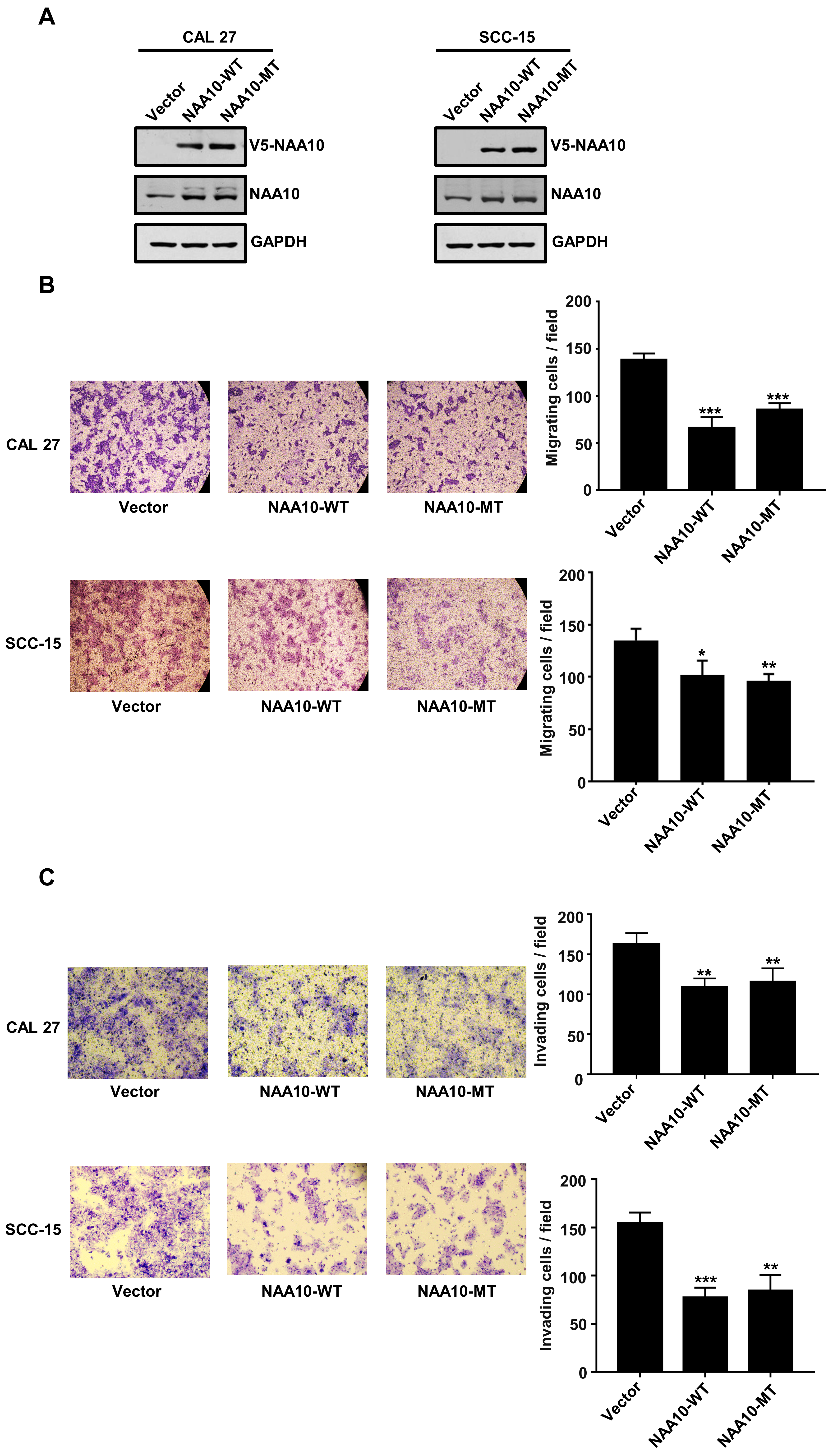
**

A, CAL 27 and SCC-15 cells were transfected with NAA10-WT or NAA10-MT, NAA10–R82A, and transfection efficiencies were determined by Western blot in CAL 27 and SCC-15 cells. B, C, Transwell assay were performed to evaluate the effect of NAA10-WT and NAA10-MT on migration (B) and invasion (C) of CAL 27 and SCC-15 cells. Each data point is representative as mean of triplicate experiments.

**Figure S3. NAA10 down-regulates the mRNA expression of Pirh2 in OSCC cells.**


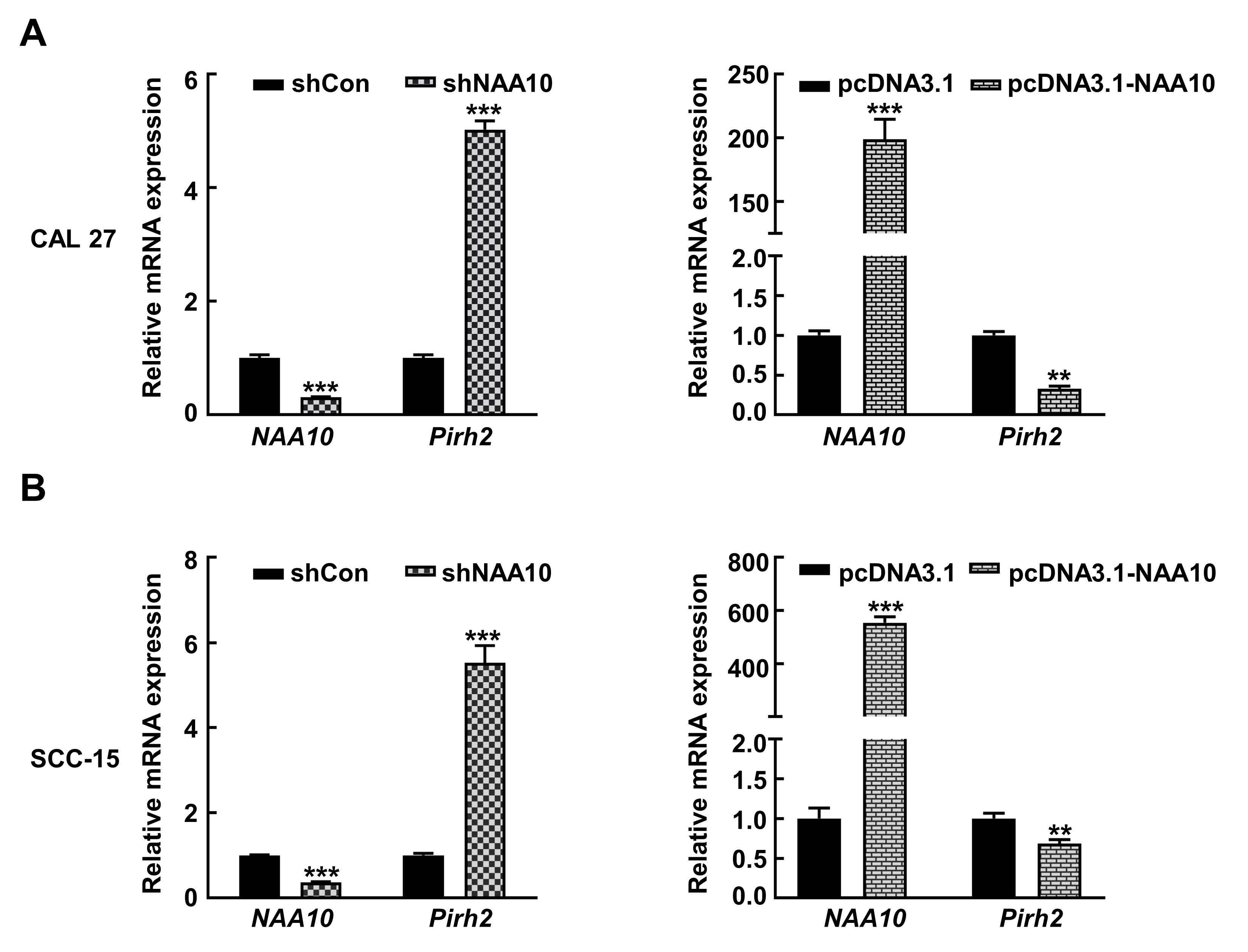


A, CAL 27 cells were transfected with NAA10-specific shRNA and plasmid of stable expression of ectopic NAA10 for 48 h. RNA samples were collected, followed by qRT–PCR analysis. B, SCC-15 cells were transfected with NAA10-specific shRNA and plasmid of stable expression of ectopic for 48 h. RNA samples were collected, followed by qRT–PCR analysis. Each data point is representative as mean of triplicate experiments.

**Figure S4. NAA10 attenuates phosphorylation of p65 in OSCC**

**
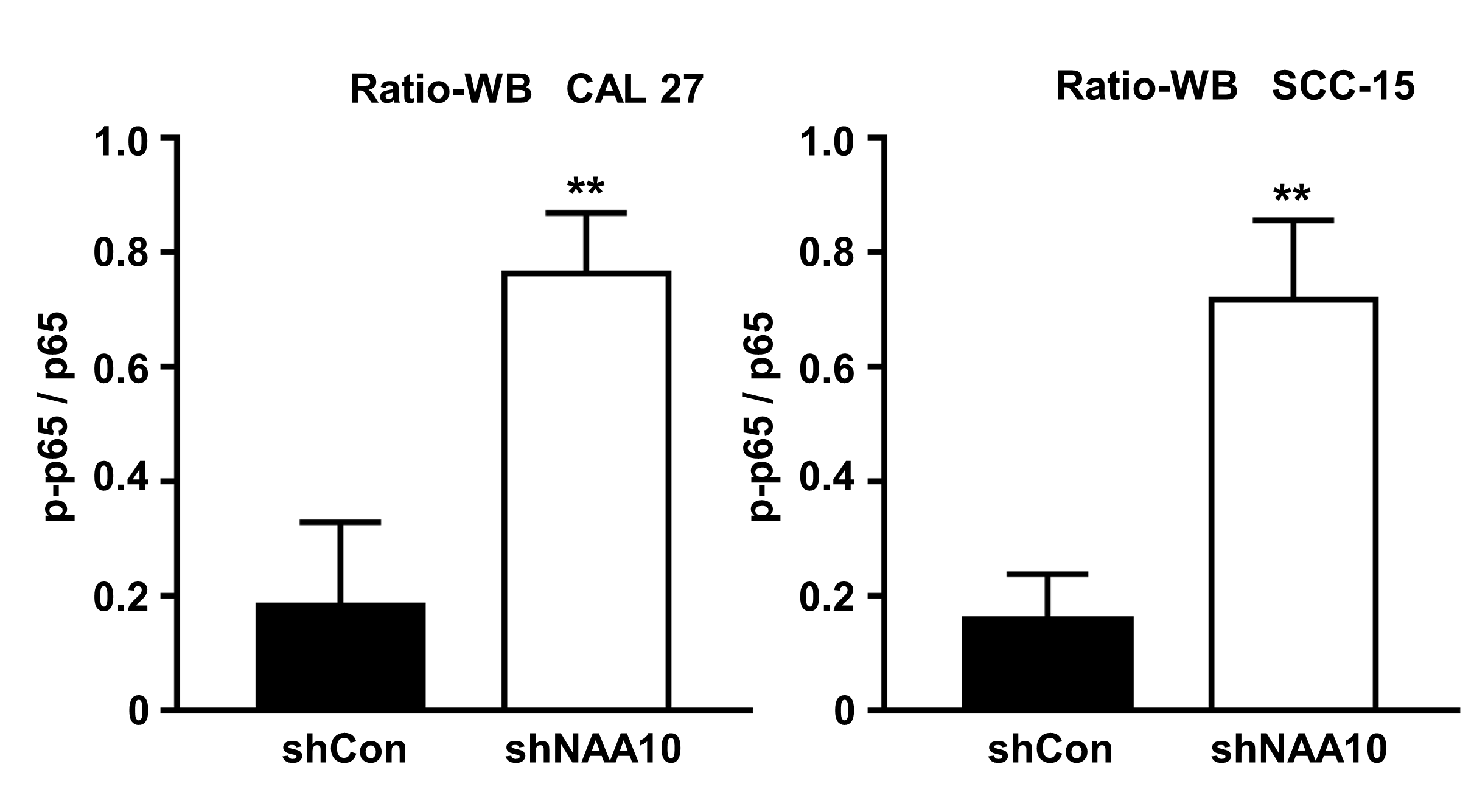
**

Ratios of p-p65 to p65 of gel blots in Figure 4F as determined by Western blot densitometry analysis. Each bar represents the mean ± SD. from three independent experiments.
